# Effects of Nordic walking on health-related quality of life in overweight individuals with Type 2 diabetes mellitus, impaired or normal glucose tolerance

**DOI:** 10.1111/j.1464-5491.2011.03348.x

**Published:** 2011-11

**Authors:** T Fritz, K Caidahl, M Osler, C G Östenson, J R Zierath, P Wändell

**Affiliations:** Center for Family and Community Medicine, Karolinska InstitutetHuddinge; * Department of Molecular Medicine and Surgery, Karolinska InstitutetStockholm, Sweden

**Keywords:** exercise, heath-related quality of life, primary health care, sleep, walking

## Abstract

**Aims:**

To assess the effects of 4 months of increased physical activity on health-related quality of life in overweight individuals with Type 2 diabetes mellitus, normal or impaired glucose tolerance.

**Methods:**

We included 212 individuals without severe physical or cardiovascular impairments aged 61 (57–64) years, with BMI of 29 (27.5–32) kg/m^2^. Numbers are median (25th–75th percentile). Subjects were stratified based on normal glucose tolerance (*n* = 128), impaired glucose tolerance (*n* = 34) or Type 2 diabetes mellitus (*n* = 50). They were randomized into either a control group (*n* = 125), who maintained unaltered habitual lifestyle, or an exercise intervention group (*n* = 87), who were directed to engage in Nordic walking with walking poles, 5 h per week over 4 months. Self-reported physical activity and health-related quality of life was assessed at the time of inclusion and after 4 months.

**Results:**

Baseline health-related quality of life of this study cohort was similar to, or better than, an age- and sex-matched Swedish population sample, for 12 of 13 scales. Quality of sleep and BMI were improved for participants with normal glucose tolerance after 4 months of Nordic walking, with little or no musculoskeletal pain as compared with control subjects. No correlation was evident between improved quality of sleep and improved BMI.

**Conclusions:**

Quality of sleep improved in the group with normal glucose tolerance following 4 months of Nordic walking. BMI reduction did not account for this improvement. Nordic walking can be introduced in a primary health care setting as a low-cost mode of exercise that promotes weight loss and improved health satisfaction.

## Introduction

A sedentary lifestyle has become a major threat to public health worldwide. Physical inactivity predisposes individuals to obesity and Type 2 diabetes mellitus and often leads to premature death. People diagnosed with Type 2 diabetes are typically middle-aged or older, with a sedentary lifestyle. Daily exercise has been considered a cornerstone in the lifestyle management of Type 2 diabetes and numerous studies have provided evidence for reduced mortality and risk of cardiovascular disease in physically active people, irrespective of the diabetic state [1–4]. Exercise intervention promotes beneficial outcomes and positively influences morbidity and mortality even in middle-aged participants [5–7]. Moreover, regular walking, combined with reduced caloric intake, has a diabetes-preventive effect in individuals with impaired glucose tolerance [8–10].

Promoting a physically active daily life should have high priority in primary healthcare counselling. A proactive intervention by primary care physicians could be a valuable approach to reduce premature cardiovascular morbidity and mortality and to delay, or even prevent, the development of impaired glucose tolerance and Type 2 diabetes. However, an older sedentary population with Type 2 diabetes is more susceptible to painful conditions of the musculoskeletal system [11]. Introducing physical activity as part of a daily regimen for the treatment of Type 2 diabetes must therefore be initiated with caution. Moreover, attention should be given to quality-of-life aspects in assessing the benefits of an exercise programme on an older, sedentary population with Type 2 diabetes.

The aim of this study was to determine if increasing daily physical activity, in the form of Nordic walking with walking poles, would influence health-related quality of life in overweight individuals with normal glucose tolerance, impaired glucose tolerance or Type 2 diabetes.

## Subjects and methods

### Patient recruitment

This investigation was a randomized, controlled study. Recruitment was achieved through newspaper advertisements and personal letters of invitation to 447 former participants in the Stockholm Diabetes Prevention Program (SDPP) [12], living in the catchment area of the primary healthcare centre of Gustavsberg, a suburb outside Stockholm, Sweden. The study spanned three consecutive summer seasons (2006–2008) and occurred between May and September. New participants were recruited each year. Two hundred and twelve individuals were included (118 females and 94 males). Ninety participants were recruited by advertisements and 122 were former SDPP participants. The participants were stratified for state of glucose tolerance (normal, impaired glucose tolerance and Type 2 diabetes) and subsequently randomized to either a control or exercise-intervention group. Each year, new entries into the study were randomized into control or intervention groups. For the randomization procedure, blinded labels with the participants’ names were drawn from a box and assigned to either the control or intervention group. Subjects randomized to the control group were invited to participate in the intervention group the following year. The subjects who joined the study in both control (first entry) and intervention (second entry) groups are represented only once in this work, as control subjects, skewing the control/exercise group numbers. The control group consisted of 125/212 individuals. The intervention group consisted of 87/212 individuals. Written informed consent was obtained from all participants. The study was approved by the Ethics Committee of Karolinska Institutet, Stockholm.

### Biochemistry and anthropometry

At the time of inclusion and after 4 months, an oral glucose tolerance test was performed. Plasma glucose was first determined in the fasting state, prior to the ingestion of 75 g of glucose in water solution, and again after 2 h, and calculated as the mean of two capillary blood samples. We used a portable HemoCue B-Glucose analyser (Ängelholm, Sweden) for this purpose [13]. Glucose tolerance was classified as either normal glucose tolerance < 8.9, impaired (impaired glucose tolerance) 8.9–12.1 or Type 2 diabetes ≥ 12.2 mmol/l, according to the 2-h level after one oral glucose tolerance test. Of the 212 subjects, 50 were classified as ‘Type 2 diabetes’, 34 as ‘impaired glucose tolerance’ and 128 as ‘normal glucose tolerance’. The mean duration (sd) of diabetes was 5 (4) years for subjects with Type 2 diabetes. Subjects who were previously diagnosed with diabetes were classified as Type 2 diabetes regardless of the oral glucose tolerance test outcome on inclusion in this study.

Body weight and height were assessed at the time of inclusion and after 4 months. BMI was calculated by the formula body weight (kg), divided by height (m) squared. A BMI of 18.5–24.9 kg/m^2^ was considered normal, 25–29.9 kg/m^2^ overweight and ≥ 30 kg/m^2^ obese. Systolic and diastolic blood pressures were determined in the seated position by the use of a Speidell and Keller tonometer (Jungingen, Germany). All measurements were made in the morning, in conjunction with the oral glucose tolerance test.

### Inclusion criteria

Inclusion criteria were as follows: age 45–69 years, BMI > 25 kg/m^2^ and, for people with Type 2 diabetes, HbA_1c_ between 57 and 78 mmol/mol [International Federation of Clinical Chemistry (IFCC) standard], comparable with 7.4–9.3% [National Glycohemoglobin Standardization Program (NGSP) standard]. Exclusion criteria were physical impairment, symptoms of angina pectoris (chest pain on physical strain), atrial fibrillation determined by electrocardiogram systolic or diastolic blood pressure > 160 or > 100 mmHg, respectively, and insulin treatment.

### Assessment of physical activity and intervention protocol

Exercise in this study was unsupervised. Self-reported exercise was recorded at baseline and after 4 months. The participants were asked to estimate their physical activity level during the 6 months prior to the study and during study participation using a visual analogue scale to rank the frequency of physical activity from 0 mm (none) to 100 mm (intensive daily activity).

The participants in the exercise group were instructed to increase their weekly level of physical activity by 5 h of Nordic walking for 4 months. Nordic walking is a unique fitness technique that utilizes walking poles to involve the upper body in the exercise, in addition to providing extra support. Participants received verbal instructions for Nordic walking by a physiologist/personal trainer. The exercise group participants were provided with a diary and asked to record date and number of minutes for each bout of Nordic walking. To compare self-reported physical activity with an objective assessment, 25 participants—11 control subjects and 14 intervention participants—agreed to wear a uniaxial accelerometer on the hip, for 7 days during waking hours (ActiGraph model GT1M; ActiGraph, Pensacola, FL, USA). This type of accelerometer records physical activity as total activity counts per min and min per day of inactivity, low, moderate or vigorous activity and is considered a valid and reliable tool for measuring physical activity in adults [14]. No instructions regarding eating habits or nutrition were provided and no dietary intervention was administered. The participants in the control group were directed to continue their habitual daily activity.

### Health-related quality of life assessment

A quality-of-life questionnaire was provided at the time of inclusion and after 4 months. The Swedish health-related quality of life questionnaire (SWED-QUAL) [15], adapted from the Medical Outcomes Study [16], was used. Medical Outcomes Study assessments are generic (as opposed to disease specific) health-related quality of life questionnaires that present results as a health profile. The 66 items of the SWED-QUAL provide the basis for 13 scales that represent the following aspects of health-related quality of life: physical functioning, satisfaction with physical health, pain, role limitation attributable to physical health, role limitation attributable to emotional health, positive affect, negative affect, cognitive functioning, sleep, general health perception, family functioning, marital functioning and sexual functioning. The multi-item scales are set between 0 and 100 points, where 0 indicates the lowest possible score and 100 the highest. Inter-group differences of the SWED-QUAL results are expressed in terms of statistical significance. Effect size is a complementary method for identifying minimally important differences of patient-reported outcomes. The minimally important difference is defined as the smallest change in a patient-reported outcome that is perceived by the patient as beneficial. The effect size is defined by the formula: effect size = (M_treatment_–M_comparison_)/SD_pooled_, M = ‘mean’ i.e. ‘M treatment’ = mean value after treatment/intervention. ‘M comparison’ = mean value before treatment/intervention. An effect size of 0.2 is considered small, 0.5 moderate and 0.8 is a large change [17]. The SWED-QUAL has been used in previous studies including patients with Type 2 diabetes [18,19]. A random sample of 2500 Swedish men and women, aged 18–85 years, has previously answered the SWED-QUAL. Health status and anthropometric data are not known for this Swedish population sample. The SWED-QUAL results of each study participant were matched, and compared with, the aggregated results of all Swedish population sample individuals of the same age and sex.

### Statistical methods

The original power calculation, based on the assumed differences in SWED-QUAL scales of 0 vs. 5 between the control and intervention groups, with α = 0.05 and power = 0.8, indicated that 63 participants would be required in each group. With the skewed randomization, explained above, a power = 0.8 would be obtained by 77/54 participants. In the group with normal glucose tolerance, we included 75/53 individuals ( = power 0.7957). The study was underpowered for participants with impaired glucose tolerance and those with Type 2 diabetes.

Stata statistical software (StataCorp., College Station, TX, USA) was used to calculate differences. The score for each scale was calculated as the median (25th–75th percentile) of the items (questions) included in each of the 13 scales. Differences within and between groups were calculated by non-parametric tests: Wilcoxon's signed-rank test for within-group comparisons and Wilcoxon's rank-sum test for between-group comparisons. Sex differences between groups were calculated by χ^2^-test. *P*-values < 0.05 were considered statistically significant. Spearman's rho was determined for the assessment of correlation.

## Results

Baseline data in Table 1 are presented separately for participants with normal glucose tolerance, impaired glucose tolerance and Type 2 diabetes. The 212 participants were 61 (57–64) years of age, with a BMI of 29 (27.5–32) kg/m^2^. Numbers are median (25th–75th percentile). Fifty-three per cent of the study participants were overweight (BMI 25.0–29.9 kg/m^2^) and 47% were obese (BMI ≥ 30 kg/m^2^). ANOVA reflected a disease-state dependency for baseline levels of HbA_1c_ (*P* < 0.001), systolic blood pressure (*P* = 0.019) and BMI (*p* = 0.0024). Diastolic blood pressure levels were, however, similar between groups of varying glucose tolerance (*P* = 0.85). The 50 participants with Type 2 diabetes reported baseline health-related quality of life scores at the same level as the 128 participants with normal glucose tolerance, for all 13 SWED-QUAL scales, when compared with Wilcoxon's rank-sum test (statistics not shown). Of the 50 participants classified as Type 2 diabetes, 45 were previously known to have Type 2 diabetes and 21 were taking anti-diabetic medication. Of the remaining five individuals classified as Type 2 diabetes, two were again classified as Type 2 diabetes according to the oral glucose tolerance test at the end of the study. Two were classified as having impaired glucose tolerance and one as having normal glucose tolerance according to this second oral glucose tolerance test. Ten participants withdrew from the study prior to the conclusion. The principle of last-observation-carried-forward was applied in those cases and their initial results were thus included in the data analysis.

**Table 1:** Baseline characteristics of participants with normal glucose tolerance, impaired glucose tolerance and Type 2 diabetes by control and intervention groups*

	NGT Control group	NGT Intervention group	*P*	IGTControl group	IGT Intervention group	*P*	Type 2 diabetes Control group	Type 2 diabetes Intervention group	*P*
*n*	75	53		20	14		30	20	
Age, years	61 (55–64)	60 (57–64)	0.9555	62.5 (59.5–64)	60 (56–63)	0.1698	60.5 (58–64)	63 (59–64)	0.6261
Sex, male/female %†	36/64	38/62	0.770	45/55	36/64	0.195	67/33	65/35	0.765
BMI, kg/m^2^	29 (27–31)	29 (27–31)	0.7663	30.5 (28–34)	31.3 (28–38)	0.6602	29.5 (28–34)	30.5 (27.5–34)	0.8265
HbA_1c_‡									
mmol/mol	38 (36–41)	37 (36–40)	0.1604	40 (40–42)	41 (37–34)	0.9718	50 (45–54)	53 (47–60)	4164
%	5.7 (5.5–5.9)	5.6 (5.5–5.8)	0.1604	5.8 (5.8–6.0)	5.9 (5.6–6.1)	0.9577	6.7 (6.2–7.1)	7.0 (6.4–7.7)	0.38280.
SBP, mmHg	140 (130–150)	140 (130–145)	0.7363	135 (132.5–150)	140 (130–155)	0.5138	145 (135–150)	145 (132.5–152.5)	0.9046
DBP, mmHg	85 (80–90)	85 (80–90)	0.8593	85 (80–90)	85 (75–90)	0.8577	85 (75–90)	85 (77.5–90)	0.5368
Physical activity estimate by VAS, mm	50 (20–60)	35 (20–50)	0.0267	35 (20–50)	26.5 (10–50)	0.5425	40 (20–50)	30 (30–60)	0.4892

Values are median (25th–75th percentile).

* *P* for intervention/control group difference by Wilcoxon's rank-sum test.

† *P* for sex distribution by χ^2^-test.

‡ Percentage [National Glycohemoglobin Standardization Program (NGSP) standard] and mmol/mol [International Congress of Clinical Chemistry and Laboratory Medicine (IFCC) standard].

DBP, diastolic blood pressure; IGT, impaired glucose tolerance; NGT, normal glucose tolerance; SBP, systolic blood pressure; VAS, visual analogue scale.

Following a 4-month period of either sustained (control group) or increased physical activity (Nordic walking, 5 h per week), BMI was reduced by –1.0 (–1.0 to 0.0) kg/m^2^ in the intervention group with normal glucose tolerance and by 0.0 (–1.0 to 1.0) kg/m^2^ in the control group with normal glucose tolerance (*P* = 0.0019). BMI did not change significantly in the groups with impaired glucose tolerance or Type 2 diabetes (Table 2).

**Table 2:** SWED-QUAL intention-to-treat analysis of changes after 4 months (ΔT4) of Nordic walking; data for participants with (a) normal glucose tolerance (NGT), (b) impaired glucose tolerance (IGT) and (c) Type 2 diabetes (T2DM), by control and intervention groups*

(a)								

	NGT	ΔT4	NGT	ΔT4	*P*‡	NGT	SPS compared with control + intervention	*P*§
								
	Control, *n* = 75	NA	Intervention, *n* = 53	NA	NA	All, *n* = 128	*n* = 128	1.000
Age, years	61 (55–64)	NA	60 (57–64)	NA	0.9555	60.5 (55–64)	NA	NA
Sex, male/female %	36/64	NA	38/62	NA	0.770	37/63	37/63	1.000
BMI, kg/m^2^†	29 (27–31)	0 (−1 to 1)	29 (27–31)	−1 (−1 to 0)	**0.0019**	29/27–31)	Unknown	NA
Physical functioning	95.3 (85.7–95.3)	0 (0:4.7)	95.3 (85.7–95.3)	0 (0–4.7)	0.7188	95.3 (85.7–95.3)	86.4 (81.7–88.7)	**< 0.0001**
Satisfaction with physical health	67 (33–67)	0 (0–33)	67 (33–67)	0 (0–33)	0.4143	67 (33–67)	65.9 (60–70.6)	**0.0030**
Pain	79.3 (64–100)	0 (−9.7 to 15.1)	79.3 (64.2–100)	0 (0–6.8)	0.1876	79.3 (64.1–100)	77.2 (74–82.2)	0.6799
Role limitation attributable to physical health	77.7 (44.3–100)	0 (−11 to 22.3)	78 (55.7–100)	0 (0–22)	0.3541	78 (50–100)	62.2 (60.5–71.2)	**0.0180**
Role limitation attributable to emotional health	100 (55.7–100)	0 (0–22)	89 (66.7–100)	0 (−11 to 11)	0.2018	94.5 (55.7–100)	74.3 (68.2–81.8)	0.0794
Positive affect	87.5 (75–95.8)	0 (−4.2 to 4.2)	87.5 (70.8–95.8)	0 (−8.3 to 12.5)	0.4218	87.5 (72.9–95.8)	76.1 (70.5–81.2)	**0.0003**
Negative affect	83.3 (62.5–95.8)	4.2 (0–12.5)	87.5 (62.5–95.8)	0 (−4.2 to 8.4)	0.2234	87.5 (62.5–95.8)	73.3 (68.3–79.7)	0.0700
Cognitive functioning	87.5 (66.7–95.8)	0 (−4.2 to 8.3)	83.3 (62.5–91.7)	0 (−4.2 to 8.4)	0.7666	83.3 (66.7–95.8)	74.6 (69.7–79.7)	**0.0099**
Sleep	82.1 (60.7–96.4)	0 (−10.7 to 3.6)	75 (46.4–92.9)	3.6 (–3.6 to 14.3)	**0.0091**	78.6 (57.1–94.7)	67.1 (62.8–74.2)	**0.0089**
General health perception	87.5 (75–93.8)	0 (0–6.2)	81.3 (65.6–93.8)	3.2 (0–12.5)	**0.0302**	84.4 (71.9–93.8)	73.6 (69.9–78.8)	**0.0005**
Family functioning	91.8 (73–100)	0 (0–0)	100 (79.3–100)	0 (0–6.3)	0.5179	93.8 (73–100)	84.3 (79.3–88.1)	0.0899
Marital functioning	83.3 (66.7–100)	0 (−4.1 to 4.2)	95.8 (70.8–100)	0 (−12.5 to 8.3)	0.8978	85.4 (68.8–100)	83.1 (77.4–88.3)	0.6352
Sexual functioning	90 (60–100)	0 (−5 to 5)	90 (65–100)	0 (0–0)	0.6014	90 (62.5–100)	64.8 (58.7–72.5)	**< 0.0001**
Physical activity estimated by VAS, mm	50 (20–60)	0 (−10 to 10)	35 (20–50)	30 (10–40)	**< 0.0001**	NA	NA	NA

(b)								

	IGT	ΔT4	IGT	ΔT4	*P*‡	IGT	SPS compared with control + intervention	*P*§
								
	Control, *n* = 20	NA	Intervention, *n* = 14	NA	NA	All, *n* = 34	*n* = 34	1.000
Age, years	62.5 (59.5–64)	NA	60 (56–63)	NA	0.1698	61.5 (58–64)	NA	NA
Sex, male/female %	45/55	NA	36/64	0.195	NA	41/59	41/59	1.000
BMI, kg/m^2^†	30.5 (28–34)	0 (−1 to 0)	31.3 (28–38)	0 (–1 to 0.5)	0.4301	30.8 (28–34)	Unknown	NA
Physical functioning	95.3 (85.7–95.3)	0 (0–4.7)	92.95 (85.7–95.3)	2.3 (0–4.7)	0.6196	95.3 (85.7–95.3)	84.9 (82.1–87.9)	**0.0122**
Satisfaction with physical health	67 (33–67)	0 (0–0)	67 (33–67)	0 (0–33)	0.5876	67 (33–67)	63 (60–70.6)	0.2415
Pain	79.3 (61.1–100)	0 (−0.1 to 20.7)	89.7 (69.7–100)	0 (0–11.3)	0.9710	79.3 (64–100)	76.7 (74–81.8)	0.3879
Role limitation attributable to physical health	78 (44.3–100)	0 (0–16.5)	61.2 (22–100)	5.5 (0–33.3)	0.6470	78 (33–100)	62.2 (62–67.8)	0.6753
Role limitation attributable to emotional health	100 (77.9–100)	0 (−5.5 to 0)	67 (33–100)	0 (0–33)	0.2550	100 (67–100)	74.9 (70.8–81.8)	0.2701
Positive affect	91.7 (83.3–97.9)	0 (−4.2 to 4.2	83.4 (45.8–95.8)	0 (−4.2 to 4.2)	0.9859	91.7 (79.2–95.8)	77.1 (70.6–81.2)	0.0588
Negative affect	87.5 (79.2–100)	2.1 (−6.2 to 12.5)	81.3 (50–95.8)	6.3 (0–37.5)	0.2115	85.4 (70.8–100)	74 (70.6–80.2)	0.1741
Cognitive functioning	93.8 (75–100)	0 (−4.2 to 6.3)	83.4 (62.5–100)	0 (−12.5 to 8.3)	0.5310	91.7 (70.8–100)	75.8 (70.2–79.7)	**0.0205**
Sleep	82.2 (60.7–94.7)	−1.8 (−9.0 to 7.1)	73.2 (57.1–89.3)	0 (−7.2 to 10.7)	0.5389	76.8 (57.1–92.9)	67.7 (60.2–75.7)	0.1303
General health perception	78.1 (64.1–90.7)	6.2 (0–17.2)	86.0 (56.3–96.9)	3.1 (0–12.5)	0.3081	84.4 (59.4–93.8)	74.5 (70.1–77.9)	0.2415
Family functioning	100 (88.7–100)	0 (0–0)	92.8 (79.3–100)	0 (–6.2 to 0)	0.7431	100 (79.3–100)	84.9 (83–87.5)	**0.0049**
Marital functioning	97.9 (75–100)	0 (0–4.2)	85.4 (54.2–100)	4.2 (0–16.7)	0.2452	87.5 (70.8–100)	83.7 (77.1–87.8)	0.4622
Sexual functioning	100 (75–100)	0 (0–0)	92.5 (45–100)	−2.5 (–10:0)	0.1752	95 (65–100)	64.1 (58.7–72.2)	**0.0039**
Physical activity estimated by VAS, mm	35 (20–50)	8.5 (0–20)	26.5 (10–50)	20 (8.5–50)	0.0784	NA	NA	NA

(c)								

	T2DM	ΔT4	T2DM	ΔT4	*P*‡	T2DM	SPS compared with control + intervention	*P*§
								
	Control, *n* = 30	NA	Intervention, *n* = 20	NA	NA	All, *n* = 50	*n* = 50	1.000
Age, years	60.5 (58–64)	NA	63 (59–64)	NA	0.6261	62 (58–64)	NA	NA
Sex, male/female %	67/33	NA	65/35	NA	0.765	66/34	66/34	1.000
BMI, kg/m^2^†	29.5 (28–34)	0 (−1 to 0)	30.5 (27.5–34)	0 (−1 to 0)	0.1867	30 (28–34)	Unknown	NA
Physical functioning	90.6 (81–95.3)	0 (−4.6 to 4.7)	90.6 (74.2–100)	0 (0–11.5)	0.5057	90.6 (76.4–95.3)	83.5 (81.4–86.4)	**0.0441**
Satisfaction with physical health	67 (33–67)	0 (0–0)	50 (33–67)	0 (0–33.5)	**0.0332**	67 (33–67)	62.5 (58.3–66.7)	0.0566
Pain	100 (73.7–100)	0 (0–4.1)	84.8 (52.9–100)	0 (−6.9 to 11.2)	0.8450	92.4 (62.7–100)	76.7 (74.6–80.1)	0.1975
Role limitation attributable to physical health	67 (44.3–100)	0 (0–22.3)	78 (50–100)	0 (0–0)	0.3203	67 (44.3–100)	62.2 (60.7–67.3)	0.2487
Role limitation attributable to emotional health	67 (44.3–100)	0 (−11 to 33)	100 (38.7–100)	0 (0–0)	0.6142	83.5 (44.3–100)	73.4 (66.1–76.5)	0.9884
Positive affect	83.3 (75–95.8)	−4.2 (−12.5 to 12.5)	87.5 (66.7–95.8)	0 (−8.3 to 0)	0.9603	83.3 (66.7–95.8)	73.6 (69.8–80.5)	0.0604
Negative affect	81.3 (62.5–91.7)	2.9 (0–16.7)	83.4 (50–100)	0 (−2.1 to 12.5)	0.7950	81.3 (54.2–100)	73.2 (68.3–78.1)	0.3566
Cognitive functioning	83.3 (58.3–95.8)	0 (−8.3–8.3)	77.1 (33.3–93.8)	0 (0–8.3)	0.1915	81.7 (58.3–95.8)	72.5 (69.4–82.5)	0.7318
Sleep	75 (64.3–96.4)	−5.3 (−14.3 to 7.2)	69.7 (51.8–85.7)	5.3 (−3.5 to 16.1)	**0.0312**	75 (60.7–96.4)	69.8 (60.2–76.3)	0.4901
General health perception	82.9 (65.6–90.6)	0 (−9.3 to 6.2)	75 (59.4–89.1)	3.1 (−3.2 to 11.0)	0.4567	81.3 (65.6–90.6)	72.8 (70.9–78.2)	0.1410
Family functioning	80.3 (73–100)	0 (0–6.3)	89.7 (57.4–100)	0 (0–27)	0.3930	83.4 (60.5–100)	84.7 (79.3–87.5)	0.2949
Marital functioning	77.1 (62.5–95.8)	0 (−8.4 to 9.4)	75 (58.3–97.9)	0 (−6.3 to 6.3)	0.8090	75 (58.3–95.8)	83.7 (77.4–87.8)	0.0703
Sexual functioning	85 (55–95)	0 (−3.8 to 5)	85 (70–95)	0 (−10 to 0)	0.2781	85 (70–95)	69.2 (59.3–75.8)	**0.0207**
Physical activity estimated by VAS, mm	40 (20–50)	0 (−1 to 15)	30 (30–60)	24 (10–40)	**0.0047**	NA	NA	NA

Data are median (25th–75th percentile).

* Baseline data for the entire NGT, IGT, and T2DM groups, respectively, are compared with the results of sex- and age-matched individuals from a healthy Swedish population sample (SPS) who have previously answered the SWED-QUAL questionnaire.

† BMI for Swedish population sample subjects not available.

‡ *P* for intervention effect by Wilcoxon's rank-sum test.

§ *P* for comparison between study participants and Swedish population sample by Wilcoxon's signed-rank test and *P* for sex distribution by χ^2^-test.

NA, not available; VAS, visual analogue scale.Bold numbers signify statistically significant differences, i.e. P < 0.05.

Visual analogue scale estimates of physical activity increased significantly in the intervention groups with normal glucose tolerance and Type 2 diabetes, by 30 (10–40) and 24 (10–40) mm, respectively, median,(25th–75th percentile), as shown in Table 2. The calculated effect sizes were 1.0 and 0.8. Walking diaries were obtained from 91% of the participants in the intervention group and 78% of the participants with normal glucose tolerance reported ≥ 80% (4 h/week) of prescribed Nordic walking. Corresponding figures for participants with impaired glucose tolerance and Type 2 diabetes were 67 and 50%, respectively. Median values (25th–75th percentile) of hours per week of Nordic walking were 4.7 (4.1–5.2), 4.6 (3.7–6.0) and 3.8 (3.1–4.7) for the groups with normal glucose tolerance, impaired glucose tolerance and Type 2 diabetes, respectively. ANOVA did not indicate any significant difference between the three groups (*P* = 0.1468). Comparison by Wilcoxon's rank-sum test showed a significant difference between participants with normal glucose tolerance and those with Type 2 diabetes (*P* = 0.0218), whereas comparisons between participants with normal–impaired glucose tolerance and impaired glucose tolerance–Type 2 diabetes showed no significant differences in hours of Nordic walking per week (*P* = 0.8702 and 0.1557, respectively).

Total counts per min, recorded by accelerometers, were 348 (220–528) for the control group (*n* = 11) and 466 (348–623) for the intervention group (*n* = 14), *P* < 0.001, Wilcoxon rank-sum test. Data are median (25th–75th percentile).

Quality of life was assessed with the SWED-QUAL questionnaire before and after 4 months of control or exercise intervention. Results of the intention-to-treat analysis of the SWED-QUAL are presented in Table 2, parallel with corresponding data from an age- and sex-matched Swedish population sample. At baseline, the group with normal glucose tolerance including both control and intervention participants scored significantly higher for eight SWED-QUAL scales [physical functioning (effect size = 0.4), satisfaction with physical functioning (0.4), role limitation because of physical health (0.2), positive affect (0.2), cognitive functioning (0.2), sleep (0.2), general health ([0.3) and sexual functioning (0.4)], compared with the Swedish population sample. For the remaining five scales, the baseline scores did not differ between the group with normal glucose tolerance and the Swedish population sample. The group with impaired glucose tolerance, including both control and intervention participants, scored significantly higher than the Swedish population sample for physical functioning (0.4), cognitive functioning (0.3), family life (0.6) and sexual functioning (0.5). For the other nine scales, there were no significant differences between participants with impaired glucose tolerance and the Swedish population sample. The group with Type 2 diabetes scored significantly higher for sexual functioning (0.3) than their Swedish population sample counterparts.

Quality of sleep and general health improved significantly in the normal glucose tolerance intervention group, compared with the normal glucose tolerance control group; effect size 0.4 and 0.1, respectively. In the Type 2 diabetes intervention group, satisfaction with physical functioning and sleep improved significantly; effect size 0.7 and 0.5, respectively. Correlation analysis showed no significant relationship between change of quality of sleep and change of BMI (Spearman's rho –0.12).

## Discussion

The findings of this study provide evidence that overweight Swedish individuals with normal glucose tolerance, impaired glucose tolerance or Type 2 diabetes report health-related quality of life at the same level as, or better than, an age- and sex-matched Swedish population sample. The participants in this study did not suffer from any severe physical or cardiovascular impairment that limited daily activity. Moreover, their blood pressure was within, or near normal range and glucose metabolic control was within the acceptable range for the participants with Type 2 diabetes (Table 1). Other studies have reported impaired health-related quality of life in people with Type 2 diabetes [20]. The most prominent factors that negatively affected health-related quality of life in people with Type 2 diabetes were macrovascular disease (coronary heart disease and stroke), psychiatric disorders (depression) and musculoskeletal disorders. In the absence of co-morbidity or diabetes complications, Type 2 diabetes does not directly impair health-related quality of life. It is noteworthy that the participants with normal glucose tolerance in our study scored significantly higher than the Swedish population sample cohort for eight SWED-QUAL scales, whereas the participants with impaired glucose tolerance scored significantly higher than the Swedish population sample cohort for four scales and the participants with Type 2 diabetes for one scale. This might imply that health-related quality of life is impaired by a worsened state of glucose tolerance. However, the number of participants with impaired glucose tolerance and with Type 2 diabetes in this study was rather low compared with the group with normal glucose tolerance. This result should therefore be interpreted with caution.

Although quality of sleep at baseline was at the same level, or better, in our study group compared with the Swedish population sample, this variable improved significantly after 4 months of increased physical activity, whereas it tended to worsen in the control group. The control group with Type 2 diabetes reported a pronounced worsening of quality of sleep. We have no satisfactory explanation for this. There were no indications of a worsened state of diabetes during the time of study that might otherwise have offered an explanation. BMI was significantly lowered in the intervention group, but there was no correlation between improved quality of sleep and lowered BMI. An increase of physical activity may therefore have a positive effect on the quality of sleep.

A report on the treatment of insomnia in adult Swedish citizens has recently been published by the Swedish Council on Health Technology Assessment [21]. Insomnia, defined as difficulty in falling asleep or early awakening more than three times per week, was reported by 24% of study participants. Moreover, 11% reported impaired daytime quality of life as a result of disturbed night-time sleep. Furthermore, a questionnaire focusing on insomnia was randomly distributed to Swedish primary care physicians. The responding physicians (*n* = 352) reported seeing two or more patients per week who requested care for insomnia, more than half of whom were over 65 years of age. Medication for sleeping disorders was prescribed for approximately 8% of adult Swedish citizens in 2008 and to elderly people, often for long-term use. There is concern that the persistent use of certain hypnotic drugs may lead to problems of adverse effects and addiction over time. Thus, Nordic walking and other modes of exercise may provide a less costly and safer alternative to medication as a means of alleviating some sleep disorders. The Swedish Council on Health Technology Assessment report concludes that the evidence for physical activity and other alternative methods for treating insomnia are insufficient and therefore further research is warranted.

Quality of sleep appears to be a sensitive marker of holistic well-being, possibly reflecting the influence of somatic disease and various factors of psychosocial stress. Individuals with Type 2 diabetes whose medical health deteriorated over 4 years have shown deteriorating quality of sleep [22]. A study of psychological distress identified insomnia as one psychological stress factor contributing to the increased risk of developing pre-diabetes and Type 2 diabetes in Swedish middle-aged men, but not in women [12]. A reduced risk of sleep disorders in men and women who participated in physical activity at least once a week, and in men who walked at a brisk pace for more than six blocks daily has been reported [23]. Studies measuring sleep physiology in individuals with or without daily exercise have been contradictory, as assessed in a meta-analytic review. Total sleep time and slow-wave sleep seemed to increase as a result of exercise, whereas rapid eye movement sleep and sleep onset latency was decreased [24]. The associations between short sleep duration and increased prevalence of Type 2 diabetes and hypertension have been described [25,26]. It has been suggested that the common mechanism may be an over-secretion of adrenocorticotrophic hormone and cortisol, and that activation of the hypothalamic–pituitary-adrenal axis in these patients may play a role [24,25]. Hypothalamic–pituitary-adrenal axis activation is regarded as an effect of various stress factors, and hypothalamic–pituitary-adrenal hyperactivity has been suggested to promote the development of insulin resistance, central obesity, dyslipidaemia, hypertension and Type 2 diabetes [27].

An association of excess weight and impaired health-related quality of life has been described [28,29]. The authors provide evidence that physical aspects of health-related quality of life are negatively affected by excess weight or obesity. The results of our study do not indicate that overweight per se, in the absence of other disabling conditions, impairs health-related quality of life.

The participants with Type 2 diabetes did not report increased pain in comparison with Swedish population sample counterparts (Table 2). This finding is highly relevant, as painful conditions of the musculoskeletal system are more prevalent in people with Type 2 diabetes than in those without Type 2 diabetes [11]. Musculoskeletal pain was a primary reason for subject withdrawal from a Dutch study of a medical fitness programme for patients with Type 2 diabetes [30]. Reported pain did not increase in exercise participants with normal glucose tolerance, impaired glucose tolerance or Type 2 diabetes in our study. No participant left the study for reasons involving musculoskeletal pain. Nordic walking may thus be a safe mode of introductory exercise, even for individuals with Type 2 diabetes.

## Limitations of this study

Four months’ intervention is potentially too brief for measurable effects to occur on some of the aspects of health-related quality of life under study. Long-term studies of similar exercise intensity and frequency are warranted to elucidate whether Nordic walking may be a sustainable mode of exercise, and whether it produces a more pronounced effect on health-related quality of life than in this brief intervention study.

The daily physical activity of participants in this study, prior to entry and during the study, was not uniform or controlled or matched. Self-reported level of physical activity at baseline varied considerably. This factor may have diminished the perceived exercise effects on health-related quality of life. The addition of Nordic walking may not have a substantial effect on quality of life to a person who was moderately physically active before inclusion in this study. In future studies of this kind, basal exercise exclusion criteria will be considered.

Exercise was not controlled and self-reporting of daily exercise, as carried out in this study, is not as accurate as supervised activities. Moreover, the visual analogue scale, utilized for estimating the frequency of daily physical activity, has not been validated as a measure of physical activity. In addition, some physical exercise normally undertaken may have been replaced by Nordic walking, thus diminishing the intended increase of physical activity during the time of study.

The numbers of participants with impaired glucose tolerance and Type 2 diabetes were too low to allow conclusions of statistical significance for these groups. The results of this study, indicating lower SWED-QUAL scores for people with impaired glucose tolerance and Type 2 diabetes, compared with participants with normal glucose tolerance must therefore be interpreted with caution.

The fact that more than half of the participants had previously been involved in the SDPP could theoretically have created some bias in our findings. The SDPP was not, however, an interventional study on the individual level and comprised no elements of lifestyle intervention. Moreover, the SWED-QUAL questionnaire was not used in the SDPP survey. Awareness of being overweight was an expressed concern and reason for the majority of individuals to participate in this study. It would seem unlikely that the former participation in the SDPP could somehow influence the results of this study.

Baseline classification of normal glucose tolerance, impaired glucose tolerance or Type 2 diabetes was based on one oral glucose tolerance test only. The diagnosis of asymptomatic diabetes in clinical practice would require the confirmation by a second oral glucose tolerance test. For the 45 participants with a diagnosis of Type 2 diabetes established prior to the time of inclusion in this study, a second oral glucose tolerance test would be redundant. Five participants were classified as ‘previously unknown Type 2 diabetes’ and, for two individuals in this group, the diagnosis was confirmed by a second oral glucose tolerance test at the end of the study. The remaining three individuals were classified as ‘impaired glucose tolerance’ (2) and ‘normal glucose tolerance’ (1) after the second oral glucose tolerance test.

## Strengths of this study

This study was undertaken in a primary care setting. It can serve as an example of how exercise could be introduced to selected patients who might profit from a more physically active lifestyle. The participants were responsible for their own exercise schedules and the study did not involve costly supervision of exercise sessions. The exercise participants received instructions from a personal trainer for Nordic walking at baseline and one supportive telephone call after 2 months from an assistant nurse.

Although not a very strenuous mode of exercise, Nordic walking is more strenuous than brisk walking as the use of walking poles also activates the muscles of the upper limbs. It may therefore suit people who are unaccustomed to exercising. Our experience was that Nordic walking did not cause adverse reactions of musculoskeletal pain.

Three different means of reporting physical activity were used in this study: visual analogue scales, exercise diaries and accelerometers. All methods indicated that the intervention group was more physically active than the control group. Although the intensity of exercise was not well established, we can reasonably assume that the self-reported amount of exercise has been a rather accurate account of actual exercise performed, based on the subjects who also used accelerometer reporting.

## Future research

The implementation of sustainable, regular exercise habits is a challenging undertaking. Exercise on prescription is presently practised in Swedish primary health care, and has been shown to promote increased self-reported physical exercise and improved quality of life [31]. Our results imply that a small increment of regular physical activity does not impair health-related quality of life. In our study, health-related quality of life tended to improve following exercise intervention. This experience could be used in clinical efforts of motivating patients to assume a more physically active life, a factor of utmost importance in health counselling of people with excess weight, disturbed glucose metabolism, hypertension and other cardiovascular risk factors. Introducing regular exercise to individuals unaccustomed to exercise training or suffering from cardiovascular disease should be done with care, taking into account the risks of cardiovascular or musculoskeletal complications.

Future studies of health-related quality of life in relation to physical activity should involve supervised exercise and participants with a somewhat uniform, and low, level of habitual exercise habits at inclusion. Elucidating whether people with impaired glucose tolerance or Type 2 diabetes are less responsive to the effects of regular exercise is clinically relevant and sufficient numbers of participants with normal glucose tolerance and Type 2 diabetes should be included.

## Abbreviations

SDPP: Stockholm Diabetes Prevention Program
SWED-QUAL: Swedish health-related quality of life questionnaire
